# Medical Items

**Published:** 1898-06

**Authors:** 


### MEDICAL ITEMS.

How about this? "The health of a young widow always improves when her physician marries."—*Ex.*

It is said that an inventive genius of Kentucky has constructed a sewing-machine for the use of surgeons in sewing up wounds.

Dr. Marion Hull, of Athens, Ga., has located in Atlanta. He will be associated with Dr. J. S. Todd at the latter's office on Marietta street.

Dr. Michael Hoke has removed his office from the Grand Opera House to the residence office of the late Dr. Hugh Hagan on Peachtree street.

The following physicians are occupying beautiful new quarters in the English-American building: Drs. L. H. Jones, R. B. Ridley, M. A. Purse, A. G. Hobbs.

Now is the time for medical colleges to advertise. The Journal has a large circulation and it will pay any college to put an advertisement in our pages. Send for rates.

Dr. Wm. Owens has just returned to Atlanta from a trip abroad. While away he devoted himself to the study of the eye, ear, nose and throat. The doctor will make Atlanta his home.

We would call attention to the progressive character of our Atlanta medical institutions, whose advertisements can be seen on the pages of this journal. All of them are well equipped and modern in every respect.

AGAIN it is announced that the University Medical College and Bellevue Medical College, New York, have united. An effort was made to combine these schools a year ago, but without success. We trust that the present plans for consolidation will be carried out.

---

THE PROPER SPIRIT.—Convalescent (dictating): "Please say to Mrs. Jackson that I thank her, not alone for the brandy peaches that she so kindly sent me, but also for the spirit in which they were sent."—*Ex.*

---

A MAN in the car was telling how good his doctor was. "Clever?" said he. "Well, I should say he was. The other day I called him in when I had swallowed five cents. He said if the coin was not counterfeit it would pass, and made me cough up two dollars."—*Medical Age.*

---

DR. M. B. HUTCHINS.—At the end of the session of 1897–8 Dr. M. B. Hutchins resigned his position as Clinical Lecturer on Skin Diseases and Syphilis in the Atlanta Medical College, for reasons satisfactory to himself. This resignation was accepted at his "own request."

---

WE extend our heartfelt sympathy to Dr. V. O. Hardon in the loss of his most estimable wife. In the bloom of womanhood death chose her as its victim. She was a woman beloved and admired by all who knew her, and many charitable institutions will miss her ever-ready aid.

---

*Mrs. McLubberty*—Here's some pills, Murty, that Mrs. Hogan was afther sindin' over for yez. She says dey'll aither kill or cure yez.

*McLubberty* (who is ill)—Begorra, did she say which dey would do foorst?—*Puck.*

A BROOKLYN landlord offers a house and lot rent free to the first family among his tenants in which twins are born. To the first family in which triplets are born he will present a house and lot. —*Ex.*

It was Mark Twain, we believe, who said that twins in a household were equal to a riot and triplets equivalent to an insurrection. If this is true our enterprising Brooklyn friend seems to be hunting trouble.

---

THE one sign of malignant disease of the uterus which should always be investigated, and especially so when it occurs at or near the menopause, is hemorrhage. We may say, I think, that in all cases in which the menstrual period becomes prolonged, the flow more profuse, or the interval shortened, the most rigid examination, no matter what the condition or age of the patient may be, is demanded. In all cases which I have observed bleeding has been the earliest symptom.—*L. G. Baldwin.*

---

THERE has been much discussion as to the best time of the day for the performance of operations. In all cases in which much fear is present, the early morning hours should be selected, as, since the patient has slept, he will not be compelled to spend a great part of the day hungry and alarmed, with each succeeding hour increasing his dread. Operations late in the afternoon possess the advantage that, as night soon comes on, the patients are more likely to fall asleep and spend a quiet night, yet we know that this result is often a problematic one, as post-operative restlessness and sometimes jactitation often prevent sleep for hours after operations.—*International Journal of Surgery.*

---

A PHYSICIAN should not presume upon the time and patience of his colleagues by reading a paper composed of truisms, some facts borrowed from text-books, and much padding. Such exhibitions contribute to international ill-feeling and to personal disdainment. No more should he report procedures based on alleged chemic or physiologic experiments which he is not ready and able to demonstrate by chemic or physiologic tests. Above all, he assiduously should refrain from announcing papers that he does not anticipate being able to present. This latter procedure has already been done to death. If he will take the results of his honest work and intelligent thought, it matters not whether they be based on experimentation or observation, he may be assured of kindly reception and courteous attention.—*Medical Record.*

---

IF you were to take an eminently practical boy and school him into the superficial, sentimental, emotional and dependent habits of the average girl, with the ordinary attendants of a corset, tight and high-heeled shoes and indoor training, and insufficient clothing, and let him live on deoxygenated air, with no hope except to get married, and not allow him to purchase even as much as a railroad ticket for himself, never have a pocket in his clothes, spend hours daily curling his hair and preparing to spend a frivolous evening, etc., he would develop into a veritable hysterical nonentity, capable of producing only his kind.—*Dr. Lucinda H. Corr.*

---

THE *St. Louis Medical and Surgical Journal* of December, 1897, contains an article on appendicitis by Dr. A. C. Bernays, of St. Louis, in which he reports a series of seventy cases without a death—all but two or three operated upon quite early in the course of the disease. He concludes: "I believe that when it comes squarely to the consciousness of the general practitioner that modern surgery, in the hands of experienced operators, can cure seventy acute cases of suppurative appendicitis in succession, without selection of cases, all other methods of treatment must be abandoned. And when it is further stated that the average time of confinement to the bed is less than three weeks, all room for discussion seems to vanish."—*Am. Jour. of Surg. and Gynecol.*

AT the recent meeting of the Alabama State Medical Association, it was determined to have a Jerome Cochran lecture delivered at the annual meetings by some prominent medical man in memory of the physician of that name. It was further determined to erect the proposed monument to Jerome Cochran at Montgomery instead of at Selma. In addition, it was agreed to ask the State legislature to make vaccination compulsory; that a law should be enacted giving State quarantine laws precedence over county laws, and that the State Board of Health should have charge of the movement of trains in the event of epidemics of contagious diseases. The following officers were elected: President, H. A. Moody, of Baily Springs; orator, G. C. Chapman, of Mobile; senior vice-president, S. G. Gay, of Selma; junior vice-president, S. H. Lowry, of Huntsville; secretary, G. R. Waller, of Montgomery; treasurer, H. G. Perry, of Greenville. Mobile was chosen as the next place of meeting.—*Phila. Med. Journal.*

---

DR. DAVID W. YANDELL, of Louisville, Kentucky, died May 3. Dr. Yandell was born in Tennessee in 1826. He was not only preeminent as a surgeon, but as a speaker and raconteur was unsurpassed. Dr. Yandell was the surgeon who attended Albert Sidney Johnston at Shiloh, and many a time had Dr. Yandell been heard to tell how he importuned the great Confederate leader, who was regarded by many as the military genius of the war, to submit to treatment and to allow others to look after the fighting for the time being. Those who knew David W. Yandell in his prime can hardly realize that for the past five years he had been mentally and physically a wreck. First his health declined, and then his mental faculties weakened, and ever since, until death, he was a confirmed invalid. He was tall of stature, with massive frame and magnificent presence. Whether seen in an ordinary crowd or in an assembly of great men, David W. Yandell was always easily recognized as a man cast in no ordinary mould. He was great as a surgeon, useful as a citizen, and delightful as a friend; and well may his profession, his country, and the wide circle of friends whom he had drawn about him mourn the death of this princely character.—*Nashville American.*

---

DR. JOHN GUITERAS, professor of pathology in the University of Pennsylvania and an eminent yellow fever expert, has been instructed by the Surgeon-General of the United States Army to proceed to Tampa, Florida, to act as medical adviser to the commander of the army which it is expected will invade Cuba. Relative to the dangers which may beset troops in Cuba, and the precautions which should be adopted, the following statement is attributed to Dr. Guiteras: "It is possible to prevent the infection of military garrisons, though whether it can be done in a campaign remains to be seen. Yellow fever is circumscribed within certain areas, and if it is possible to keep the troops away from those areas there will be little danger of infection. Contrary to the prevailing idea, altitude does not govern the disease. There are no extremely high altitudes in Cuba, and yet there are places where there is no yellow fever. In some places on the coast the disease is not to be found. As a general rule, the more important the town, the greater its commercial activity, the more infected it is. Yet a congregation of people in the interior could not originate yellow fever. The cities where the disease prevails are infected because they are permanently inhabited by a crowd. Still, the disease may be carried to a garrison from an infected town. To guard against this the troops must be placed by themselves, in uninfected places, and they must not communicate with infected places. Then, too, no depot of suppplies should be placed in an infected port. This is, of course, a desideratum that it may be difficult to obtain, for strategic reasons. Ideal conditions are not always possible in a military campaign. Whether or not yellow fever can be kept from the troops depends entirely upon whether these plans can be carried out."—*Phila. Med. Journal.*

